# Shugoshin Prevents Dissociation of Cohesin from Centromeres During Mitosis in Vertebrate Cells

**DOI:** 10.1371/journal.pbio.0030086

**Published:** 2005-03-01

**Authors:** Barry E McGuinness, Toru Hirota, Nobuaki R Kudo, Jan-Michael Peters, Kim Nasmyth

**Affiliations:** **1**Research Institute of Molecular PathologyViennaAustria; Stowers Institute for Medical ResearchUnited States of America

## Abstract

Cohesion between sister chromatids is essential for their bi-orientation on mitotic spindles. It is mediated by a multisubunit complex called cohesin. In yeast, proteolytic cleavage of cohesin's α kleisin subunit at the onset of anaphase removes cohesin from both centromeres and chromosome arms and thus triggers sister chromatid separation. In animal cells, most cohesin is removed from chromosome arms during prophase via a separase-independent pathway involving phosphorylation of its Scc3-SA1/2 subunits. Cohesin at centromeres is refractory to this process and persists until metaphase, whereupon its α kleisin subunit is cleaved by separase, which is thought to trigger anaphase. What protects centromeric cohesin from the prophase pathway? Potential candidates are proteins, known as shugoshins, that are homologous to *Drosophila* MEI-S332 and yeast Sgo1 proteins, which prevent removal of meiotic cohesin complexes from centromeres at the first meiotic division. A vertebrate shugoshin-like protein associates with centromeres during prophase and disappears at the onset of anaphase. Its depletion by RNA interference causes HeLa cells to arrest in mitosis. Most chromosomes bi-orient on a metaphase plate, but precocious loss of centromeric cohesin from chromosomes is accompanied by loss of all sister chromatid cohesion, the departure of individual chromatids from the metaphase plate, and a permanent cell cycle arrest, presumably due to activation of the spindle checkpoint. Remarkably, expression of a version of Scc3-SA2 whose mitotic phosphorylation sites have been mutated to alanine alleviates the precocious loss of sister chromatid cohesion and the mitotic arrest of cells lacking shugoshin. These data suggest that shugoshin prevents phosphorylation of cohesin's Scc3-SA2 subunit at centromeres during mitosis. This ensures that cohesin persists at centromeres until activation of separase causes cleavage of its α kleisin subunit. Centromeric cohesion is one of the hallmarks of mitotic chromosomes. Our results imply that it is not an intrinsically stable property, because it can easily be destroyed by mitotic kinases, which are kept in check by shugoshin.

## Abstract 2

Shugoshin helps to keep newly replicated chromosomes together by protecting cohesins from phosphorylation, until the moment for the chromosomes to separate has arrived

## Introduction

Cohesion between sister chromatids ensures that traction of sister chromatids towards opposite poles (known as bi-orientation) generates a tug-of-war between microtubules attempting to pull sisters apart and cohesion between them resisting this. The resulting tension is thought to stabilize the attachment of kinetochores to microtubules [1]. Only when all chromosomes have come under tension and have congressed to the metaphase plate do cells destroy the connection holding sister chromatids together, which triggers the simultaneous disjunction of all sister chromatid pairs and traction of sister chromatids to opposite poles of the cell, known as anaphase.

Sister chromatid cohesion depends on a multisubunit complex called cohesin, which is composed of a heterodimer of Smc1 and Smc3 proteins associated with an α kleisin protein called Scc1 [2,3], that is in turn associated with either SA1 or SA2 variants of the Scc3 protein. These proteins create a gigantic ring structure within which DNA molecules might be entrapped [4,5]. Sister chromatid cohesion is destroyed at the metaphase-to-anaphase transition because of cleavage of cohesin's α kleisin (Scc1/Rad21) subunit by a protease called separase [6], whose activity causes cohesin to dissociate from chromatin. For most of the cell cycle, separase activity is inhibited by its phosphorylation by the Cdk1 kinase [7] and by the binding of an inhibitory chaperone called securin [8,9,10]. Separase is eventually activated by proteolysis of the Cdk1 cyclin B subunit and securin, both mediated by a ubiquitin protein ligase called the anaphase-promoting complex or cyclosome (APC/C) [11,12]. Because of the production by unattached kinetochores of an inhibitory form of the Mad2 protein, which binds an essential APC/C cofactor called Cdc20, the APC/C destroys securin and cyclin B only when all chromosomes have successfully bi-oriented [13]. This surveillance mechanism is known as the mitotic spindle checkpoint. Cells lacking any one of cohesin's four subunits fail to bi-orient chromosomes properly and arrest at least transiently in a mitotic state because of inhibition of APC/C-Cdc20 by Mad2 [14,15].

In yeast, cohesin persists at centromeres and along chromosome arms until the onset of anaphase, whereupon it is removed by the APC/C-separase pathway [8]. In animal cells, however, the bulk of cohesin associated with chromatin during G2 dissociates from chromosome arms but not from centromeres during prophase and prometaphase [16]. This so-called “prophase pathway” is thought to be driven not by cleavage of cohesin's α kleisin subunit but instead by hyperphosphorylation of Scc3-SA subunits [17] mediated (directly or indirectly) by the Aurora B [18] and Plk1 mitotic kinases [19]. Crucially, expression of an Scc3-SA2 subunit whose serine and threonine residues phosphorylated during mitosis have been mutated to alanine reduces the dissociation of cohesin from chromosome arms, which, as a consequence, remain more tightly associated [17]. Surprisingly, this mutation does not dramatically interfere with mitosis, presumably because the extra cohesin associated with metaphase chromosomes is efficiently removed by separase upon activation of the APC/C. The function of the prophase pathway is therefore unclear. Centromeric cohesin is not just more slowly removed by the prophase pathway because cohesin remains at centromeres during the prolonged mitotic arrest that is caused by activation of the spindle checkpoint. Under these circumstances, cohesion between chromosome arms is lost entirely, while that between sister centromeres persists [20]. Centromere-specific factors presumably protect cohesin from the prophase pathway.

An analogous process occurs during meiosis, during which cohesin along chromosome arms must be treated differently from cohesin at centromeres. Because of recombination between maternal and paternal chromatids, cohesin along chromosome arms holds homologous centromeres together and enables their bi-orientation. Cleavage of cohesin's α kleisin subunit by separase along chromosome arms destroys this connection and thereby triggers the first meiotic division [21,22]. However, cohesin at centromeres is refractory to separase during meiosis I and therefore persists until metaphase II, and these cohesin complexes are crucial for the bi-orientation of sister chromatids during the second meiotic division. Recent work has shown that a class of proteins associated with meiotic centromeres, called MEI-S332 in Drosophila melanogaster [23] but now known as shugoshins, are essential for cohesin's ability to persist at centromeres after anaphase I. Shugoshin protects cohesin from separase during meiosis I in yeast [24,25,26]. Surprisingly, shugoshins are also found at centromeres during mitosis in yeast [24,27] and D. melanogaster [28]. This observation raises the possibility that shugoshins might have an important, albeit different, function during mitosis.

We show here that a human shugoshin that is possibly orthologous to fly MEI-S332 and yeast Sgo1 proteins [24,25,26] associates with centromeres during prophase and disappears at the onset of anaphase in mitotic tissue culture cells. HeLa cells whose shugoshin has been depleted by RNA interference (RNAi) fail to retain cohesin at centromeres during mitosis, and, as a consequence, their sister chromatids separate asynchronously before anaphase can be initiated, which triggers a prolonged mitotic arrest. Remarkably, expression of nonphosphorylatable Scc3-SA2 alleviates both the precocious loss of sister chromatid cohesion and the mitotic arrest. This suggests that centromeric shugoshin prevents phosphorylation of cohesin's Scc3-SA2 subunit and thereby protects the cohesion between sister centromeres that is essential for mitosis.

## Results

### The Sgo1/MEI-S332 Protein Accumulates at Centromeres as Cells Enter Mitosis but Disappears during Anaphase

The human and mouse genomes each contain a single gene (*hsSgo1* and *mmSgo1,* respectively) that encodes proteins with a similar structure and weak homology to fungal Sgo1 proteins and MEI-S332 from D. melanogaster. Analysis of cDNAs from various databases as well as RT-PCR products from mRNA obtained from HeLa cells and mouse testis suggest that variable splicing may give rise to several types of protein (Figure S1). To detect these proteins, we raised two antibodies against peptides from the N and C termini that are common to all human splice variants.

**Figure 1:**
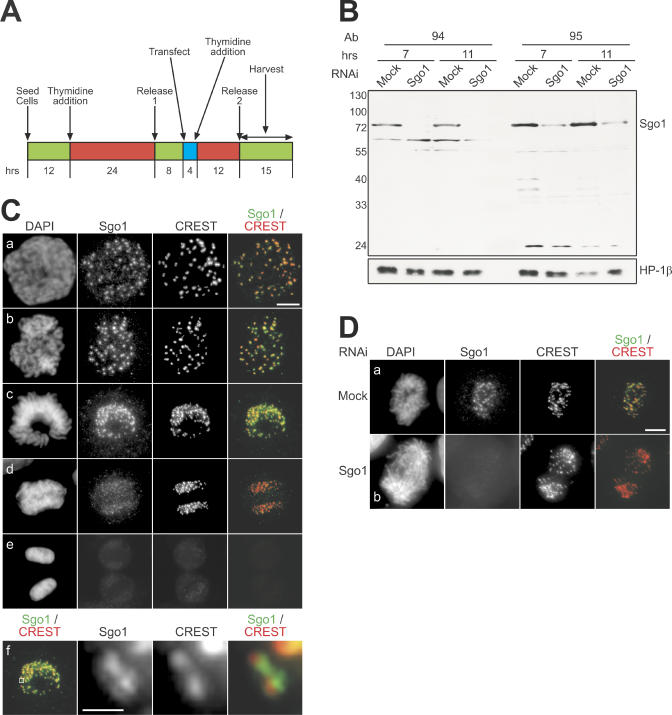
Centromeric Enrichment of Sgo1 in Mitosis (A) Schematic overview of cell synchronisation and transfection procedure. (B) Characterization of Sgo1 antibodies. Affinity-purified Sgo1 antibodies raised against two different regions of Sgo1 recognize the same 72-kDa protein by Western blotting. Synchronised HeLa cells were transfected with water (Mock) or Sgo1 siRNA. Cells were harvested 7 and 11 h after release from thymidine block, and chromatin fractions were resolved by SDS-PAGE and probed with antibodies 94 and 95. A major band at 72 kDa was detected by both antibodies, which disappeared or was greatly reduced upon Sgo1 knockdown. The blot was reprobed with HP-1β antibody as a loading control. (C) Localisation of Sgo1. Cells were costained for Sgo1 with antibody 94 (shown in green), and with CREST antiserum (shown in red). DNA was counterstained with DAPI. Five different stages of mitosis are shown: (a) prophase, (b) prometaphase, (c) metaphase, (d) anaphase, and (e) telophase. Bar = 10 μm. (f) A single pair of CREST labelled kinetochores are enlarged, showing two adjacent Sgo1 foci as commonly observed for late stage metaphases. Bar = 1 μm. (D) In situ immunofluorescence of (a) mock-treated or (b) Sgo1 siRNA-transfected HeLa cells, demonstrating the disappearance of the Sgo1 signal following siRNA treatment. The latter shows the typical in situ appearance of an Sgo1-depletion arrested cell (see Figures 3 and 6). Sgo1 was stained with antibody 94 (shown in green), and CREST is shown in red. DNA was counterstained with DAPI.

To evaluate the specificity of affinity-purified antibodies, we tested whether immunofluorescence signals or bands on Western blots were abolished when Sgo1 mRNA was depleted by transfecting cells with small interfering RNAs (siRNAs) (Figure 1). Because most subsequent experiments addressed Sgo1's role during mitosis, siRNA transfection in HeLa cells was combined with a double thymidine block and release to synchronise cells (Figure 1A). Cells were transfected with siRNAs 8 h after release from the first thymidine block, and 4 h later a second block was imposed by repeat addition of thymidine. After a further 12 h, cells were released from the second block and samples taken at specific times thereafter. Under these circumstances, most cells complete S phase within 7 h of the second release and soon thereafter enter mitosis. Both affinity-purified antibodies detected a 72-kDa protein from a chromatin fraction that was depleted by treatment with an Sgo1-specific siRNA but not by a mock treatment (Figure 1B). This size is consistent with an approximately 60-kDa protein predicted from the longest of the cDNAs analysed.

**Figure 2:**
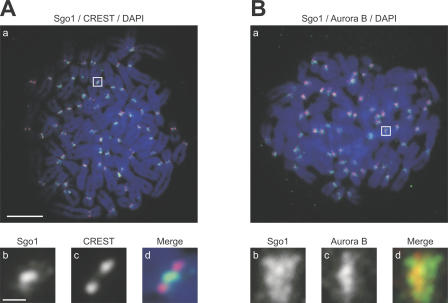
Immunofluorescent Staining of Chromosome Spreads from HeLa Cells Chromosome spreads were stained for Sgo1 with antibody 94 (shown in green), and counterstained with (A) CREST antiserum (bar = 10 μm), and (B) antibody to Aurora B, (both shown in red). DNA was stained with DAPI (shown in blue). Enlarged views show a single Sgo1 focus located between two sister CREST dots (A), and overlapping with Aurora B focus (B). Bar = 1 μm [applies to images b–d in (A) and (B)].

In situ immunofluorescence of cycling HeLa cells suggested that Sgo1 accumulates at centromeres during mitosis (see Figure 1C). Very similar staining patterns were observed with antibodies against both peptides (Figure S2). The centromeric staining was eliminated in most cells by siRNA depletion (see Figure 1D). Only 16% of all mitotic cells remained positive for Sgo1 staining following siRNA treatment, compared to 99% in mock-treated cells. Sgo1 started to form foci on chromosomes as cells entered prophase (see Figure 1C, part a). During prometaphase, Sgo1 was concentrated, usually as a single focus, on the inner side of the twin (sister kinetochore) structures stained by a CREST (calcinosis, Raynaud's phenomenon, esophageal dysmotility, sclerodactyly, telangiectasias) antiserum (see Figures 1C, part b, and 2A) thought to detect the kinetochore proteins CENP-A/B or C, and Sgo1 overlapped with and covered a slightly larger area than Aurora B during prometaphase (Figure 2B). During metaphase, two Sgo1 foci overlapping with or adjacent to the two CREST signals were observed at many kinetochores (Figure 1C, part f). Centromere-associated Sgo1 was much less abundant during anaphase, but it could nevertheless still be faintly detected adjacent to kinetochores at the leading edge of chromatids (see Figure 1C, part d). No Sgo1 foci could be detected in telophase cells (see Figure 1C, part e). A similar pattern was observed after transfection with a gene expressing a Sgo1-GFP fusion protein (E. Watrin and J.-M. Peters, personal communication). The distribution of Sgo1 during mitosis in HeLa cells resembles that of MEI-S332, its presumptive orthologue in D. melanogaster [28].

### Sgo1 Is Required to Maintain Sister Chromatid Cohesion in Mitotic Cells

**Figure 3:**
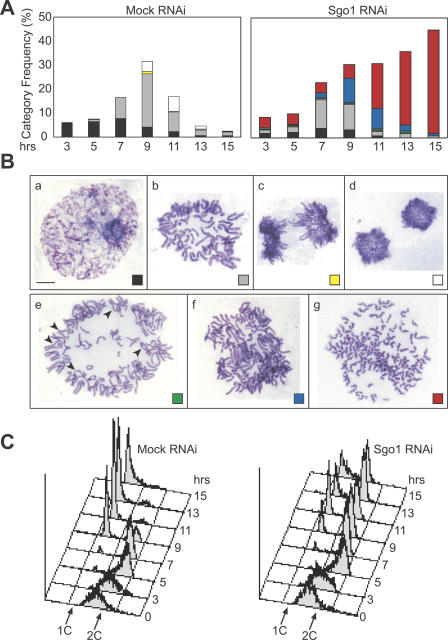
Sgo1 Depletion Causes Precocious Sister Separation and Mitotic Arrest Synchronised HeLa cells were transfected with Sgo1 siRNA or dH_2_O, harvested at 2-h intervals following release and examined by chromosome spreading and Giemsa staining. 100 cells were scored for mitotic index, and 100 mitotic cells were classified into seven categories based on chromosome configuration as exemplified in (B) for each time point. (A) The frequency of each category of mitotic cell is given as a percentage of total cell numbers, such that the sum of each column represents the mitotic index. (B) Representative pictures of seven categories of mitotic cells. Chromosome spreads: (a) prophase; (b) metaphase/metaphase-like; (c) anaphase; and (d) telophase. (e) Early phase of precocious sister disjunction. At this stage, sisters are beginning to separate and some or all presumptive sister pairs are still discernible. Arrowheads indicate chromosomes whose centromeric cohesion seems to be lost. (f) Later phases of sister chromatid separation. Sister pairs at this stage are no longer discernible, remnants of the metaphase plate are still visible, and sisters have not yet hypercondensed. (g) Scattered single chromatids. Sisters are completely separated and distributed randomly in relation to one another, individual chromatids are hypercondensed, giving a “curly” appearance. Note that chromatid separation in normal anaphases (c) are different from precocious sister chromatid separation (e–g), in that disjoined and paired chromatids coexist in the same cell. (C) A portion of the cells harvested for the analysis in Figure 3A and 3B were ethanol-fixed and their DNA content was analysed by flow cytometry.

To address the function of Sgo1 in HeLa cells, we harvested cells at 2-h intervals after release from a double thymidine block and compared cells whose Sgo1 protein had been depleted by siRNA to mock-treated cells (see Figures 1A, 1B, and 3). Sgo1 depletion had little or no effect on the kinetics of S phase completion or on entry into mitosis (see Figure 3A and 3C). At each time point, chromosomes were spread on glass slides after methanol-acetic acid fixation, and stained with Giemsa. We first measured the fraction of chromosome spreads in a mitotic state, then measured the fraction of seven different categories of mitotic chromosomes amongst mitotic cells (see Figure 3A and 3B). In mock-treated cells, the fraction of mitotic spreads peaked 9 h after release and subsequently declined. The most frequent category at early time points was prophase (see Figure 3B, part a), and the most frequent category at 9 h was metaphase (see Figure 3B, part b). Early-anaphase cells (see Figure 3B, part c) were very rare, whereas telophase cells (see Figure 3B, part d) accumulated later than metaphase cells. Sister chromatids were either tightly associated as during metaphase or fully separated into two equal-sized clusters, as during anaphase or telophase.

Sgo1-depleted cells accumulated in mitosis with similar kinetics, but many failed subsequently to exit from a mitotic state, with the result that nearly 50% of the cells had accumulated in a mitotic state 15 h after release (see Figure 3A). FACS analysis showed that around half of the cells failed to undergo cytokinesis (see Figure 3C). Many Sgo1-depleted cells accumulated at least transiently with chromosomes in a metaphase-like state, but at all time points a large number accumulated with fully condensed or even hypercondensed chromatids that had separated from their sisters (see Figure 3B, part g). At 9 h, when the frequency of metaphase cells peaked in mock-treated cultures, we observed not only a high fraction of metaphase cells but an equally high fraction of spreads in which most sister chromatid pairs had disjoined but chromatids had not yet hypercondensed (see Figure 3B, part f). Although many sisters had separated in these cells, we could also detect remnants of a metaphase plate. We also detected a small fraction of spreads in which this abnormal separation of sister chromatids had only commenced—namely, spreads in which sister chromatids were still aligned but much farther apart than during a normal metaphase (see Figure 3B, part e). These data suggest that Sgo1-depleted cells enter mitosis and align most chromosomes on a metaphase plate, but subsequently fail to undergo anaphase. They then disjoin their sister chromatids asynchronously and in a manner that is not accompanied by directed movement to opposite poles, and, finally, arrest for a prolonged period with hypercondensed, fully separated chromatids. Because the transfected siRNAs could, in principle, interfere with the RNAi machinery that might be necessary for centromeric sister chromatid cohesion [29], we tested whether other siRNAs produce a similar phenotype. Not one of 12 different siRNAs caused the rapid mitotic arrest with separated sister chromatids that is characteristic of cells treated with Sgo1 siRNAs (Figure S3).

### The Kinetics of Sister Chromatid Separation in Sgo1-Depleted Cells

**Figure 4:**
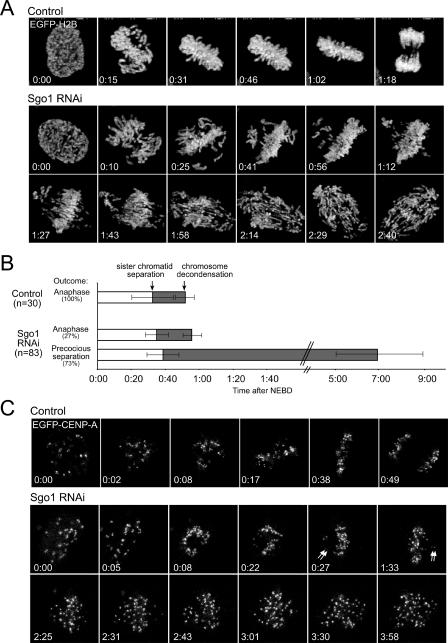
Live Cell Analysis of Sgo1-Depleted Cells (A) Synchronised HeLa cells expressing EGFP-tagged histone H2B were transfected with Sgo1 siRNA or dH_2_O as in Figure 1A, and analysed with time-lapse confocal microscopy. Stacks of 12 different z-plane images were obtained every 5 min and projected images for several time points are shown. Note that several chromosomes failed to congress to the metaphase plate, which was followed by progressive sister chromatid separation over time. Typically, a pair of disjoining sister chromatids dissociated simultaneously from the metaphase plate towards opposite poles. (B) Summary of live cell analysis. Cells prepared as in (A) were examined by time-lapse immunofluorescence microscopy. Mitotic cells were aligned on the time axis according to NEBD as determined from the loss of a defined nuclear boundary. Time from NEBD to anaphase onset/sister chromatid separation (white portion of bars) and from this point to chromosome decondensation or apoptosis (grey portion of bars) was measured. (C) To follow centromere dynamics, cells expressing EGFP-CENP-A were either mock-treated or Sgo1 siRNA transfected. Images were obtained by time-lapse confocal microscopy. Note that in cells depleted of Sgo1, dots of paired GFP-CENP-A can be found in earlier unaligned chromosomes (arrows). Single dots progressively fell apart in later phases, resulting in collapse of metaphase plate (images in lower row).

To analyse the consequences of Sgo1 depletion with greater temporal resolution, we filmed mock-treated and Sgo1-depleted HeLa cells that stably express histone H2B tagged with enhanced green-fluorescent protein (EGFP), which enabled us to observe the movement of individual chromosomes and chromatids (Figure 4). After mock treatment, all cells that entered mitosis subsequently underwent anaphase. The time from nuclear envelope breakdown (NEBD) to anaphase onset was 33 ± 9 min (average ± standard deviation) (*n* = 30). In Sgo1-depleted cells, 73% of cells that entered mitosis separated sister chromatids without undergoing anaphase. Although most chromosomes initially congressed to a metaphase plate, many were slow to do so and some never congressed at all (Figure 4A). Although 27% of mitotic cells underwent anaphase, we noticed most of these did so with lagging chromatids (unpublished data). The time from NEBD to the point at which the first sister chromatid was pulled out from the metaphase plate in Sgo1-depleted cells was 39 ± 12 min (*n* = 60), which is only slightly longer than the time taken by mock-treated cells to commence anaphase or the time taken by the minority of Sgo1-depleted cells that underwent anaphase (34 ± 7 min; *n* = 23). Cells that disjoined sister chromatids precociously remained in a mitotic state with condensed but fully separated chromatids for a prolonged period (6 h 20 min ± 2 h 3 min) before chromatids decondensed or cells underwent apoptosis. Kinetochore movements in mock-treated and Sgo1-depleted cells were also compared by filming cells expressing a CENP-A protein fused to EGFP (Figure 4C). The results confirmed that most sister kinetochore pairs congressed to a metaphase plate before splitting precociously, which led to collapse of the metaphase plate. It also confirmed the congression defect of some sister kinetochore pairs (Figure 4C, arrows). Our data imply that the highly abnormal separation of sister chromatids seen in Sgo1-depleted cells occurs at around the same time as cells would normally undergo anaphase and not as soon as cells enter mitosis. This suggests that Sgo1 may be required not to build sister chromatid cohesion during S phase, but rather to maintain sister chromatid cohesion during mitosis, a period during which most cohesin is removed from chromosome arms. Our data also show that the abnormal sister chromatid separation of Sgo1-depleted cells is not merely a response to an extended mitotic arrest.

### Sgo1 Prevents Precocious Dissociation of Cohesin from Centromeres During Mitosis

**Figure 5:**
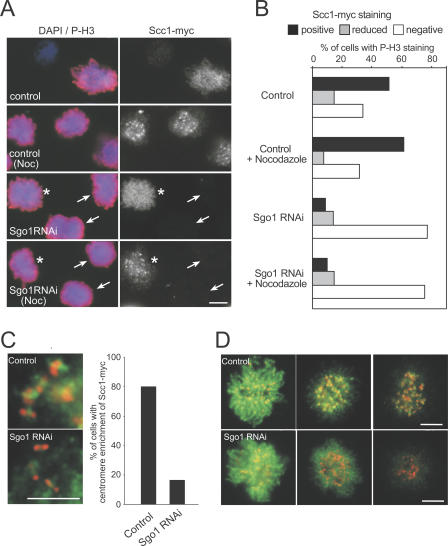
Sgo1 Is Required for Stable Association of Cohesin at Centromere (A) Synchronised HeLa cells that inducibly express Scc1-myc were transfected with control or Sgo1 siRNA and processed for immunofluorescence microscopy with or without 4-h treatment with nocodazole. Mitotic cells were spun down on glass slides and analysed for cohesin with antibodies to myc epitope (right photomicrographs). Cells were costained with P-H3 (left photomicrographs, red) to identify cells from prophase to metaphase. DNA was counterstained with DAPI (left photomicrographs, blue). Note that with Sgo1 RNAi, Scc1-myc was undetectable in the majority of P-H3 positive cells (arrows). Cells with myc staining are indicated as staining controls (asterisks). Bar = 10 μm. (B) Quantification of Scc1-myc staining. Approximately 200 cells with positive staining for P-H3 were assessed for Scc1-myc staining, which was classified as positive, reduced, or negative, as indicated. (C) Lack of centromere enrichment of cohesin in Sgo1-depleted cells. Mitotic cells were prepared as in (A), and the centromeric enrichment of Scc1-myc was analysed by immunofluorescence microscopy. Merged pictures of Scc1-myc (green) and CREST antigen (red) are shown. Approximately 200 cells were scored for each experiment. Bar = 5 μm. (D) Centromeric cohesin is not maintained in Sgo1-depleted cells. Mitotic cells were prepared and processed for immunofluorescence as in (C). Representative cells with various levels of Scc1-myc are shown. Note that centromeric staining of Scc1-myc emerges as the bulk of cohesin dissociates from chromosomes in controls (upper panels). However, in Sgo1 RNAi cells, centromeric enrichment is hardly seen at any stage of arm cohesin dissociation (lower photomicrographs). Bar = 10 μm.

To test if the abnormal separation of sister chromatids in Sgo1-depleted cells is accompanied by loss of cohesin from chromosomes, we depleted Sgo1 from synchronised HeLa cells that inducibly express a myc-tagged version of the Scc1 α kleisin cohesin subunit (Figure 5) [16]. Cells were harvested 9.5 h after release from the second thymidine block when many had already entered mitosis. After mock treatment, Scc1-myc staining along chromosomes was detectable in two-thirds of mitotic cells, which were identified by being positive for histone H3 phosphorylation (Figure 5A and 5B). Scc1-myc was typically more abundant at centromeres in such cells. This centromere enrichment was more clearly detectable (Figure 5A) if cells had been incubated in the presence of the spindle poison nocodazole for 4 h prior to harvesting, a treatment that induces a prometaphase arrest during which cohesin is completely removed from chromosome arms. Sgo1 depletion greatly reduced the fraction of mitotic cells with chromosomal Scc1-myc staining, in both the presence and the absence of nocodazole (Figure 5A and 5B). Interestingly, we noticed that Scc1-myc was rarely if ever enriched at centromeres in the few mitotic Sgo1-depleted cells whose chromosomes were still associated with Scc1-myc (Figure 5C and 5D). Importantly, Sgo1 depletion did not reduce the amount of Scc1-myc associated with prophase chromosomes (Figure S4). These results are consistent with the notion that the loss of mitotic sister chromatid cohesion caused by Sgo1 depletion is due to dissociation of cohesin from centromeres before cells initiate anaphase.

### Sgo1-Depleted Cells Arrest in a Prometaphase-Like State

**Figure 6:**
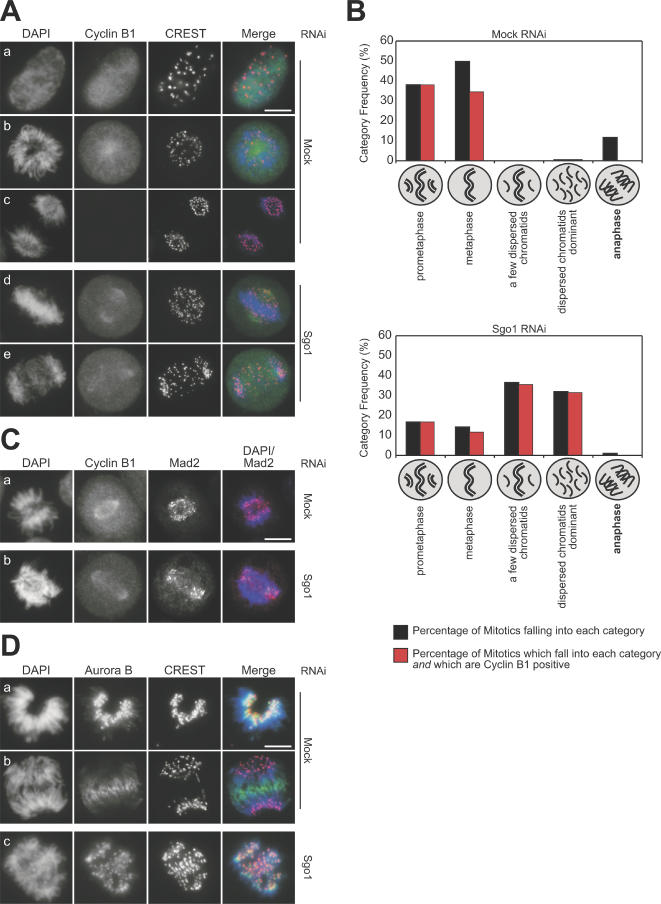
Sgo1-Depleted Cells Arrest in a Prometaphase-like State (A) Sgo1 depletion prevents destruction of cyclin B1. Synchronised HeLa cells mock-treated or transfected with Sgo1 siRNA were processed for immunofluorescence microscopy using cyclin B1 antibodies (green) and CREST sera (red) 11 h after release from the second thymidine block. DNA was visualized by DAPI staining (blue). In mock-treated cells, cyclin B1 staining in prophase (a) and in prometaphase (b) disappears as cells segregate their chromosomes upon anaphase entry (c). Sgo1-depleted cells which segregate chromosomes asynchronously and arrest in a mitotic state retain preanaphase levels of cyclin B1 (d–e). Bar = 10 μm. (B) Mitotic cells shown in (A) were classified into five categories based on DAPI-labelled chromosome configuration, and scored for presence or absence of cyclin B1 staining. Black bars represent the frequency of each category, and red bars represent the percentage of cells that fall into each category *and* are positive for cyclin B1 staining. (C) Recruitment of Mad2 in cells depleted of Sgo1. Cells prepared as in (A) were stained with cyclin B1 antibody, and counterstained with Mad2 antibody (red) and DAPI to visualize DNA (blue). (a) Prometaphase mock-transfected cells show Mad2 staining at kinetochores, which is lost as the metaphase plate assembles and disappears before cells enter anaphase (unpublished data). (b) Sgo1-transfected cells which have prematurely segregated sisters remain positive for Mad2 kinetochore staining. Bar = 10 μm. (D) Aurora B remains at centromeres in Sgo1-depleted cells. Cells prepared as in (A) were stained with Aurora B antibody (green) and counterstained with CREST antiserum (red), and DAPI to visualize DNA (blue). In prometaphase (a) mock-transfected cells, Aurora B is found at centromeres before relocalizing to the central spindle as cells enter anaphase (b). In Sgo1 transfected cells that show precocious sister separation, Aurora B remains localized at centromeres (c).

To investigate whether the loss of cohesin from centromeres is due to precocious activation of separase, we used in situ immunofluorescence to assess cyclin B1 levels and localization of Mad2 in mock-treated and Sgo1-depleted cells 11 h after their release from the second thymidine block. In mock-treated cells, cyclin B1 was concentrated within the nuclei of prophase cells and on the mitotic spindles of all prometaphase and most metaphase cells, although also throughout their cytoplasm, and was absent from anaphase or telophase cells (Figure 6A). To quantitate this result, we classified mitotic cells into five categories based on DAPI (4′,6′-diamidino-2-phenylindole) staining and scored the fraction of each category that was positive for cyclin B1 (Figure 6B). In mock-treated cells, cyclin B1 was positive in all prometaphase cells, in about 70% of metaphase cells, and in no anaphase cells. This pattern reflects destruction of cyclin B1 shortly before the onset of anaphase. In Sgo1-depleted cells, cyclin B1 was positive in all prometaphase and most metaphase cells. It was also positive in the vast majority of cells that contained metaphase plates with some dispersed chromatids and in cells whose chromatids had largely dispersed from the metaphase plate (Figure 6B). This implies that cyclin B1 destruction never occurs in mitotic Sgo1-depleted cells. This is presumably due to continued activation of the mitotic spindle checkpoint because the chromosomes of mitotic cells lacking Sgo1 always contained foci of Mad2 associated with their centromeres, which is normally seen only in prometaphase in mock-treated cells (Figure 6C and unpublished data). These data suggest that separase is not activated in Sgo1-depleted cells. Their loss of centromeric cohesin is therefore unlikely to be due to Scc1 cleavage.

Another event that normally occurs at the onset of anaphase is the disappearance of Aurora B from inner centromeres and its accumulation at the spindle midzone [30]. Aurora B does not dissociate from centromeres at any stage of the abnormal mitoses of Sgo1-depleted cells, as Aurora B was found adjacent to CREST staining not only in metaphase cells (unpublished data) but also in cells whose chromatids have dispersed from the metaphase plate (Figure 6D). A corollary of this finding is that cohesin is not required to maintain Aurora B at centromeres, contrary to a previous suggestion [15].

### Neither Plk1 Depletion nor Aurora B Inhibition Suppresses the Precocious Sister Separation in Sgo1-Depleted Cells

**Figure 7:**
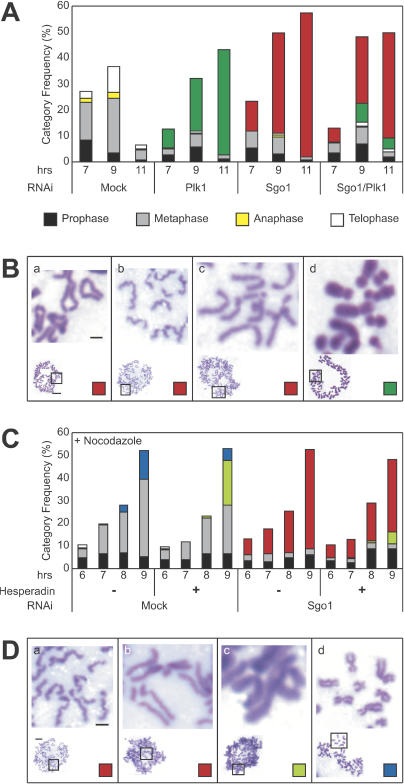
Neither Plk Depletion nor Aurora B Inhibition Suppresses the Precocious Sister Separation Seen in Sgo1-Depleted Cells (A and B) Synchronised HeLa cells were mock-treated or transfected with the indicated combination of Sgo1 and Plk1 siRNA and harvested at the time shown following release from thymidine block. Chromosomes were then spread on glass slides and examined by Giemsa staining. As in Figure 3, the percentage of mitotic cells were calculated out of 200 cells, and mitotic chromosome spreads were then further classified into one of 10 categories (*n* = 200 mitotic spreads for each time point). (A) The frequency of each category of mitotic cell (see examples in Figures 3B and 7B) is given as a percentage of total cell numbers, such that the sum of each column represents the mitotic index. (B) In addition to the seven categories in Figure 3A and 3B, the following categories (illustrated in Figure 7B) were scored: (a) sister centromeres separated, arms still cohesed; (b) scattered chromatids, same as Figure 3B, part g; (c) sisters are separated and randomly distributed in relation to one another, but are not hypercondensed; and (d) sister chromatids are cohesed along their length, and rod-shaped chromosomes are hypercondensed, as is characteristically seen with Plk1 knockdown [20]. For simplicity, the categories illustrated in Figure 3B, part e and f, and Figure 7B, part a–c, were combined into a single category representing premature sister separation (shown in red). In smaller pictures, bar = 10 μm; in enlarged portions, bar = 2 μm. (C and D) Synchronised HeLa cells were mock-treated or transfected as indicated. At 6 h after release from the second thymidine block, cells were treated with nocodazole with or without Aurora B inhibitor Hesperadin as indicated, and harvested at hourly intervals up to 3 h thereafter. Mitotic index and chromosome spreads were assessed as in (A). (C) The frequency of each category of mitotic cell (illustrated in Figures 3B and 7D) is given as a percentage of total cell numbers, such that the sum of each column represents the mitotic index. (D) The seven categories scored for Figure 3A and 3B were also scored for this experiment. (a) Scattered chromatids as in Figure 3B, part g; (b) early phase of precocious sister disjunction as in Figure 3B, part e. In addition the following categories (illustrated in Figure 7D) were also scored: (c) Chromosomes have begun to decondense prior to separation, and sister resolution is defective, characteristic of Hesperadin treatment. (d) Centromeres cohesed, and arms opened and hypercondensed, characteristic of nocodazole treatment.

Removal of cohesin from chromosome arms during prophase and prometaphase depends on the activity of mitotic kinases, including Plk1. Might this Plk1-dependent process in normal cells be prevented from attacking centromeric cohesin by Sgo1? To test this idea, we analysed whether synchronised cells depleted for both Sgo1 and Plk1 also lose sister chromatid cohesion when they enter and arrest in mitosis. Mock-treated, Plk1-depleted, Sgo1-depleted, and Sgo1- and Plk1-depleted cells were harvested at 7, 9, and 11 h after release from the thymidine block, and the state of spread chromosomes was analysed by Giemsa staining (as described in Figure 3). The mitotic index of mock-treated cells peaked at 9 h and declined by 11 h, but the mitotic indices of singly or doubly depleted cells continued to rise after 9 h, reaching around 50% by 11 h (Figure 7A). Plk1-depleted cells arrested with hypercondensed, rod-shaped chromosomes whose arms were more tightly associated than those of prometaphase or metaphase mock-treated cells (Figure 7B, part d), which is consistent with previous findings [20]. Sgo1 singly depleted cells arrested with separated chromatids, progressing via the same set of stages described in Figure 3B. Importantly, cells depleted for both Sgo1 and Plk1 also largely arrested with separated chromatids (Figure 7A). The morphology of these chromatids was, however, very different from those seen in Sgo1 singly depleted cells. Of those cells whose sister chromatids had been separated (i.e., those represented by the red bars in Figure 7A), 100% possessed short, curly (presumably coiled) separated chromatids by 11 h in a culture depleted of Sgo1 alone, as shown in Figure 7B, part b. In contrast, 80% of such cells that had been depleted for both Sgo1 and Plk1 possessed separated chromatids that were both longer and straighter, as shown in Figure 7B, part c. Only 12% possessed short, curly chromatids, while 8% possessed chromatids whose arms were still loosely associated with their sisters, as shown in Figure 7B, part a.

The fact that treatment with Plk1 siRNA dramatically changed the morphology of separated chromatids in Sgo1-depleted cells confirms that both proteins had in fact been effectively depleted. Our data therefore imply (somewhat surprisingly) that Plk1 is not necessary for the precocious separation of sister chromatids induced by Sgo1 depletion. Interestingly, Sgo1 depletion permitted not only sister centromere separation but also that along chromosome arms in cells supposedly lacking Plk1. This raises the possibility that the tight cohesion between sister chromatid arms that persists in Plk1-depleted cells depends on Sgo1.

Aurora B kinase activity has also been implicated in the removal of cohesin from chromosome arms. To address whether this kinase is responsible for the precocious loss of sister centromere cohesion in Sgo1-depleted cells, we examined whether inhibition of Aurora B by the small molecule inhibitor Hesperadin can suppress their sister chromatid separation. Because Aurora B and its yeast equivalent Ipl1 are also required to prevent cell cycle arrest of cells with defective sister chromatid cohesion [31] or cells whose microtubules cannot generate centromeric tension [32,33], it was necessary to maintain Mad2 inhibition of APC/C activity by addition of nocodazole at the same time that Hesperadin was added, 6 h after they had been released from the thymidine block. The former delays by at least 3 h the exit from mitosis of Hesperadin-treated cells [32]. Chromosomes were spread and examined by Giemsa staining after harvesting mock-treated and Sgo1-depleted cells at 6, 7, 8, and 9 h after release (Figure 7C and 7D). Addition of nocodazole caused both mock-treated and Sgo1-depleted cells to accumulate in mitosis between 6 and 9 h after release, which was largely unaffected by Hesperadin addition. Importantly, Hesperadin had only a modest, if any, effect on the precocious separation of sister chromatids in Sgo1-depleted cells (Figure 7C and 7D).

Interestingly, treatment with nocodazole had a clear effect on the arrangement of the separated sisters in Sgo1-depleted cells. By 9 h, up to 73% of mitotically arrested cells resembled the image shown in Figure 7D, part b, where single chromatids lie in the neighbourhood of their presumptive sisters. In the absence of nocodazole, less than 5% of cells fell into this category (Figure 3B, part e). This implies that when sister cohesion is prematurely lost as a result of Sgo1 depletion, spindle pulling forces are not required to separate sisters but they are required to produce the scattered chromatid effect seen in the previous experiments.

We conclude that Hesperadin cannot prevent the precocious separation of sister chromatids induced by Sgo1 depletion, at least when such cells are prevented from exiting mitosis by addition of nocodazole. In a very similar experiment in which nocodazole was omitted, Hesperadin did indeed reduce the number of mitotic cells whose sisters had separated precociously (unpublished data). We believe that this effect is probably a statistical artefact caused by the failure of Sgo1-depleted cells treated with Hesperadin to arrest in mitosis.

### The Precocious Sister Separation and Mitotic Arrest of Sgo1-Depleted Cells Is Suppressed by a Nonphosphorylatable Scc3-SA2

In a related paper, Hauf et al. [17] describe the mitosis-specific phosphorylation of 12 serine or threonine residues clustered within the C-terminal domain of cohesin's Scc3-SA2 subunit. Remarkably, a large fraction of Scc3-SA2 protein in which all 12 residues have been mutated to alanine (Scc3-SA2 12xA), which is no longer phosphorylated during mitosis, persists on chromosome arms throughout mitosis and even does so when cells are arrested for prolonged periods in a prometaphase-like state due to nocodazole treatment. The persistence of Scc3-SA2 12xA on chromosomes under these circumstances prevents loss of cohesion between sister chromatid arms in nocodazole-arrested cells, but it does not obviously interfere with mitosis in cycling cells. This implies that phosphorylation of Scc3-SA2 may be largely, if not solely, responsible for the removal of cohesin from chromosome arms during prophase and prometaphase.

**Figure 8:**
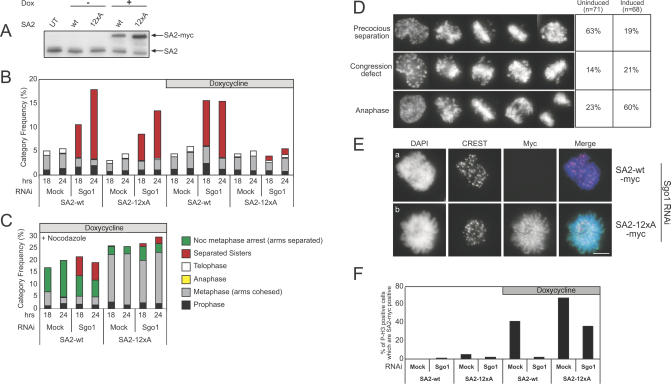
Expression of Nonphosphorylatable Scc3-SA2 Suppresses Sgo1-Depletion Phenotype (A) Total cell extracts prepared from HeLa cells that, upon doxycycline treatment, inducibly express either a wild-type (SA2-wt) or nonphosphorylatable (SA2–12xA) myc-tagged version of Scc3-SA2 were resolved by SDS-PAGE and probed with anti-SA2 antibody. The lower band represents endogenous Scc3-SA2, the upper band, myc-tagged Scc3-SA2. The first lane contains total cell extract prepared from untagged HeLa cells. (B) Expression of nonphosphorylatable Scc3-SA2 suppresses sister separation caused by Sgo1 depletion. Cycling HeLa cells carrying inducible myc-tagged versions of Scc3-SA2 were treated with (or without) doxycycline at 2 μg/ml for 72 h prior to and during transfection to induce expression, as indicated. Cells were harvested 18 h and 24 h post transfection, chromosomes were spread on glass slides and Giemsa-stained. As in Figure 7, the percentage of mitotic cells were calculated out of 200 cells, and mitotic chromosome spreads were then further classified into one of five categories (*n* = 200 mitotic spreads for each time point). The frequency of each category of mitotic cell (see examples in Figures 3B, 7B, and 7D) is given as a percentage of total cell numbers, such that the sum of each column represents the mitotic index. (C) Nonphosphorylatable Scc3-SA2-mediated suppression is still observed when combined with nocodazole arrest. The indicated treatments were repeated as outlined in (B) in the presence of nocodazole which was added to cultures 4 h posttransfection, i.e., 14 and 20 h prior to harvesting of cells. Samples were processed as in (B), with the addition of a sixth mitotic category. (D) Live cell analysis of Sgo1 depletion of nonphosphorylatable Scc3-SA2 (SA2–12xA) expressing cells. To follow chromosome behaviour, cells stably expressing EGFP-H2B and inducibly expressing SA2–12xA were used. Expression of SA2–12xA was induced 72 h prior to transfection as in (B). Cells were transfected with Sgo1 siRNA as in Figure 1A and examined by time-lapse fluorescence microscopy. Significant number of cells exit mitosis by expressing nonphosphorylatable Scc3-SA2. (E) Nonphosphorylatable SA2 (SA2–12xA) is found at centromeres even in the absence of Sgo1. HeLa cells containing either the myc-tagged wild-type (a) or SA2–12xA (b) inducible transgene were either uninduced or induced as in (B) 72 h before transfection. Transfection of Sgo1 siRNA was performed prior to the second thymidine block as in Figure 1A. At 8.5 h after the release from early S phase, mitotic cells were spun down on glass slides and analysed for cohesin localisation by immunofluorescence microscopy using antibodies to the myc epitope (shown in green). Cells were costained with CREST antiserum to label kinetochores (shown in red) DNA was counterstained with DAPI (shown in blue). (F) Quantification of SA2-myc staining. Samples similar to those described in (E) were stained with myc and P-H3 antibodies (the latter to identify cells from prophase to metaphase). Approximately 200 P-H3-positive cells were assessed for SA2-myc staining, and the percentage of cells that were both P-H3- and SA2-myc-positive was plotted. We believe that the apparent drop in the number of SA2–12xA-myc positive cells observed with depletion of Sgo1 relative to mock transfection is a statistical artefact caused by the mitotic arrest and accumulation of those cells that did not express SA2–12xA-myc (∼30%) but that were depleted of Sgo1.

We therefore analysed the effects of Sgo1 depletion in cell lines in which either wild-type SA2 or SA2 12xA protein (both tagged with nine myc epitopes) is expressed from a doxycycline-inducible promoter at levels (when induced) that are comparable to endogenous SA2 protein (see Figures 8A and S5). We conducted the experiments on cycling cells that had been treated with (or without) doxycycline for 72 h prior to transfection. Cells were then transfected either with water (mock) or with the Sgo1 siRNA and harvested 18 or 24 h later, and chromosome spreads were examined after Giemsa staining. In mock-transfected cultures, the frequency of mitotic cells remained constant (at around 4–5%) at 18 and 24 h, regardless of the presence or absence of doxycycline or whether wild-type or mutant protein was induced (Figure 8B). In Sgo1-depleted cells, the mitotic indices of cells expressing wild-type Scc3-SA2 myc increased to between 16% and 18% by 24 h; the mitotic index also increased to 14% in cells that expressed only very low levels of Scc3-SA2 12xA-myc in the absence of doxycycline. As expected, most sister chromatids had separated in both sets of these Sgo1-depleted cells. The low frequency of telophase cells suggests that these cells largely failed to undergo anaphase. Remarkably, induction of Scc3-SA2 12xA-myc with doxycycline suppressed the accumulation of mitotic cells caused by Sgo1 depletion. Moreover, only a few of the mitotic cells contained precociously separated sister chromatids, and significant numbers had clearly undergone anaphase and produced telophase cells (Figure 8B). Most metaphase cells possessed chromosomes whose sisters were cohesed at centromeres as well as along their arms. Thus, expression of nonphosphorylatable Scc3-SA2 at physiological levels suppresses both the mitotic arrest and the precocious sister chromatid separation caused by Sgo1 depletion. This indicates that loss of cohesin from centromeres in Sgo1-depleted cells may be due to (hyper-) phosphorylation of Scc3-SA2 at centromeres.

It is nevertheless possible that nonphosphorylatable Scc3-SA2 suppresses a mitotic defect of Sgo1-depleted cells responsible for their cell cycle arrest, and as a consequence these cells might not have long enough to lose sister chromatid cohesion. To address this possibility, nocodazole was added to doxycycline-induced cells 4 h after transfection (i.e., 14 or 20 h prior to harvesting), which caused both mock-treated and Sgo1-depleted cells to accumulate in mitosis (Figure 8C). In the case of the culture in which Sgo1 had been depleted and wild-type SA2 myc protein induced, a large fraction of mitotic cells (31%) had separated sister chromatids without undergoing anaphase. This fraction was much lower (9%) when SA2 12xA-myc protein had been induced. Most Sgo1-depleted mitotic cells expressing SA2 12xA-myc contained intact arm, as well as centromere, sister chromatid cohesion (Figure 8C). We conclude that SA2 12xA-myc expression suppresses loss of sister chromatid cohesion in Sgo1-depleted cells even when cells have been arrested in mitosis for many hours.

To examine more carefully whether expression of nonphosphorylatable Scc3-SA2 permits cells lacking Sgo1 to undergo anaphase, we created a new cell line that expressed histone H2B tagged with EGFP as well as doxycycline-inducible Scc3-SA2 12xA-myc. Sgo1 was depleted in induced and uninduced cells from the same cell line and the behaviour of chromosomes followed by time-lapse video microscopy (Figure 8D). Three types of mitoses were observed: those in which sister chromatid pairs first congressed to a metaphase plate but then lost cohesion before undergoing anaphase (precocious separation), those in which some chromosomes were slow to congress and failed to undergo anaphase but did not display mass sister separation, and those in which chromosomes congressed to a metaphase plate and then underwent what appeared to be a normal anaphase with unaltered kinetics. Only 23% of uninduced cells depleted for Sgo1 underwent anaphase, and 63% separated their chromatids precociously, while the rest had congression defects. Remarkably, induction of Scc3-SA2 12xA-myc increased the fraction of cells that underwent anaphase from 23% to 60% and reduced the fraction of cells that separated sisters precociously from 63% to 19%. These data suggest that expression of Scc3-SA2 12xA-myc enables cells to undergo anaphase in the absence of Sgo1.

Finally, we addressed whether the loss of mitotic phosphorylation sites on Scc3-SA2 enables cohesin to persist at centromeres as well as along chromosome arms in mitotic Sgo1-depleted cells. To do this, we analysed mitotic chromosomes from Sgo1-depleted cells expressing either Scc3-SA2-myc or Scc3-SA2 12xA-myc. Wild-type SA2 myc-tagged protein was associated with chromosomes in very few cells, while nonphosphorylatable SA2 myc tagged protein was found along the axes of all chromosomes in nearly 40% of cells (Figure 8E and 8F). Crucially, Scc3-SA2 12xA-myc was associated with centromeres as well as chromosome arms. Preventing Scc3-SA2 phosphorylation therefore enables cohesin to persist at centromeres in mitotic cells lacking Sgo1 as well as on chromosome arms in otherwise wild-type mitotic cells. This suggests that the loss of cohesin from centromeres in Sgo1-depleted cells is due to mitosis-specific phosphorylation of SA2.

## Discussion

It has long been recognized that cohesion between sister chromatids in the vicinity of centromeres has an especially important role during both meiosis and mitosis. Centromeric cohesion is special during meiosis because it completely resists destruction at the first meiotic division when dissolution of cohesion along chromosome arms triggers the resolution of chiasmata. It is special during mitosis because, unlike cohesion along chromosome arms, centromeric cohesion persists even when cells are arrested in mitosis for prolonged periods by spindle poisons. The work described in this paper suggests, somewhat surprisingly, that both of these special attributes of centromeric cohesion might be conferred by the same protein, namely Sgo1.

Recent work suggests that, in both budding and fission yeast, Sgo1 protects centromeric cohesion at meiosis I by preventing cleavage of cohesin's α kleisin subunit by separase. Surprisingly, both Sgo1 in S. cerevisiae and its probable orthologue in D. melanogaster MEI-S332 are associated with centromeres during mitosis as well as meiosis. We show here that a related protein in human cells concentrates at centromeres during mitosis and disappears from chromosomes during anaphase. Remarkably, depletion of human Sgo1 by RNAi prevents HeLa cells from completing mitosis. Though most chromosomes congress to a metaphase plate in Sgo1-depleted cells, cells never undergo anaphase and instead arrest for a prolonged period in a prometaphase-like state with high cyclin B1 levels. Our data suggest that this is caused by a catastrophic separation of sister chromatids at around the time cells should have normally undergone anaphase, and that this event occurs in the absence of APC/C activation or dissociation of Aurora B from centromeres. This precocious separation is accompanied by loss of cohesin from centromeres as well as chromosome arms and activation of the mitotic spindle checkpoint, as documented by persistent recruitment of Mad2 to kinetochores. Remarkably, expression of a version of cohesin's Scc3-SA2 subunit whose C-terminal domain can no longer be phosphorylated as cells enter mitosis (Scc3-SA2 12xA-myc) not only alleviates the precocious loss of sister chromatid cohesion of Sgo1-depleted cells but also permits a large fraction of them to complete what appears, at least superficially, to be a normal mitosis.

**Figure 9:**
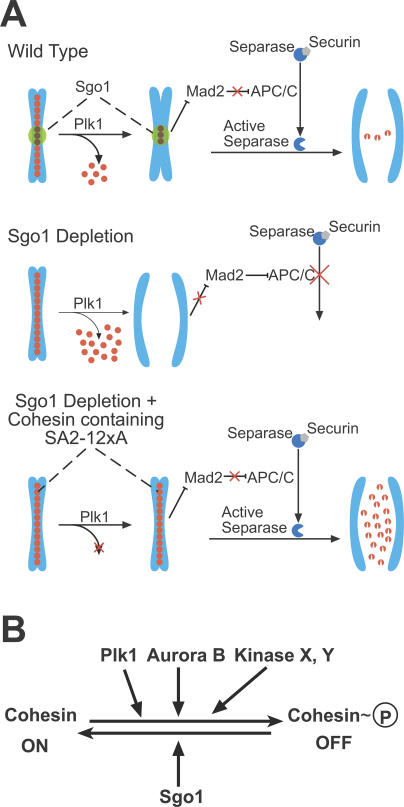
Model for Sgo1 Function during Mitosis (A) During an unperturbed mitosis (Wild Type), arm cohesin (red circles) is removed in a kinase-dependent manner during prophase/prometaphase. Sgo1 protects centromeric cohesin (brown circles) until the metaphase-anaphase transition. Once all chromosomes have successfully bi-oriented on the metaphase plate, Mad2 inhibition of the APC/C is relieved, allowing separase activation. Separase in turn removes cohesin remaining at centromeres through cleavage of the α kleisin Scc1 subunit, allowing the cell to enter anaphase. In the absence of Sgo1 (Sgo1 Depletion), cohesin is removed from the chromosome arms and at the centromere during prophase/prometaphase before chromosomes have properly bi-oriented and been attached to their full complement of spindles. Thus, Mad2 activity continues to maintain the spindle checkpoint, causing cells to arrest for a prolonged period in a prometaphase-like state with separated sister chromatids. Expression of nonphosphorylatable Scc3-SA2 (Sgo1 Depletion + Cohesin containing SA2–12xA) prevents the prophase removal of cohesin both from arms and centromeres, thus allowing cells to proceed through an apparently normal anaphase even in the absence of Sgo1 activity. (B) The phosphorylation state of cohesin may be the result of a dynamic balance in which Sgo1 somehow functions to antagonize the activity of mitotic kinases, including Plk1 and Aurora B and, potentially, other as-yet unidentified kinases (Kinase X, Y). In this model, when the direction of the reaction is artificially sent towards the hyperphosphorylated state (following Sgo1 knockdown), cohesin efficiently dissociates from chromatin; when the opposite state is favoured (e.g., as a result of Plk1 depletion) cohesin remains tightly associated with chromatin.

Our findings together with those described by Hauf et al. [17] suggest that phosphorylation of Scc3-SA2 normally triggers dissociation of most cohesin from chromosome arms during prophase and prometaphase, but is prevented from doing so at centromeres by Sgo1, which is more abundant at this location than it is along chromosome arms (Figure 9A). We suggest that in the absence of Sgo1, Scc3-SA2 is removed by the prophase pathway from centromeres as well as from chromosome arms and that this leads to the complete disjunction of sister chromatids before cells can initiate anaphase, which is meanwhile delayed by the mitotic checkpoint (Figure 9A).

The suppression of the mitotic arrest induced by Sgo1 depletion by Scc3-SA2 12xA-myc implies that it is the precocious loss of sister chromatid cohesion and not the lack of some other function of Sgo1 that is largely responsible for the pathological mitotic arrest of Sgo1-depleted cells. Our findings demonstrate for the first time that Sgo1-like proteins promote sister chromatid cohesion by regulating the activity of cohesin. This is inconsistent with the suggestion that Sgo1/MEI-S332 proteins confer a form of sister chromatid cohesion during mitosis that is distinct from that conferred by cohesin [34,35].

Salic et al. [35] have also shown very recently that Sgo1 is required to prevent precocious sister kinetochore splitting. The authors suggested that Sgo1 has an additional role in stabilizing microtubules attached to kinetochores. Their proposal, that a defect in this putative function is the primary cause of the mitotic arrest due to Sgo1 depletion, is inconsistent with our observations. In our view, the simplest, although by no means only, explanation for the mitotic arrest of Sgo1-depleted cells is that loss of sister chromatid cohesion eliminates the tension needed to stabilize the connection between microtubules and kinetochores, which in turn leads to unoccupied kinetochores that recruit Mad2 and generate a form of Mad2 that is capable of inhibiting the APC/C's destruction of securin and cyclin B. Nevertheless, our finding that 14% of Sgo1-depleted cells expressing SA2 12xA-myc had congression defects is consistent with the notion that Sgo1 might also function to regulate kinetochore-microtubule interactions.

How might Sgo1 regulate cohesin? One possibility is that Sgo1 enables cohesin to be refractory to the effects of Scc3-SA2 phosphorylation. If cohesin embraces chromatid fibres within its ring structure, then phosphorylation of Scc3-SA2's C-terminal tail might induce the cohesin ring to open, and this process might be blocked by Sgo1. Alternatively, Sgo1 might prevent cohesin's dissociation by blocking Scc3-SA2's phosphorylation in the first place or by recruiting a phosphatase that reverses its phosphorylation (Figure 9B). Antibodies capable of specifically detecting phosphorylated Scc3-SA2 would make it possible to distinguish the first from the second and third scenarios.

Several lines of evidence suggest that the process against which Sgo1 protects centromeric cohesin does not involve separase-mediated Scc1 cleavage. First, Scc3-SA2 12xA-myc's ability to suppress cohesin's dissociation both from arms in wild-type cells and from centromeres in Sgo1-depleted cells suggests that these two phenomena have a common cause. If the former is separase-independent, then so, presumably, is the latter. Second, the persistence of cyclin B1 throughout the period during which centromeric cohesion is lost in Sgo1-depleted cells implies that the APC/C is not activated and separase must therefore remain associated with its inhibitor chaperone securin. Third, cells expressing Scc3-SA2 12xA-myc proliferate fairly normally, and their timely destruction of sister chromatid cohesion at the onset of anaphase presumably involves the activation of separase by the APC/C. If separase deregulation were responsible for cohesin's precocious disappearance from centromeres in Sgo1-depleted cells, then Scc3-SA2 12xA-myc would have to suppress this defect by delaying Scc1 cleavage, which is difficult to reconcile with the timely onset of anaphase in cells expressing Scc3-SA2 12xA-myc. It is therefore likely that Sgo1 protects centromeric cohesin from different processes during mammalian mitosis and yeast meiosis, from chromosomal dissociation induced by Scc3-SA2 phosphorylation during mitosis, and from Rec8 or Scc1 cleavage during meiosis. Although different, both processes may have a key property in common, namely regulation by Plk1-mediated phosphorylation.

It is noteworthy that many shugoshins, including human Sgo1, disappear from centromeres during anaphase. This event might be triggered by their destruction at the hands of the APC/C along with securin and cyclin B [35]. This event might nevertheless assist in the dissolution of cohesion between sister centromeres at the onset of anaphase by exposing cohesin to the effects of its phosphorylation. Hyperphosphorylation of Scc3-SA2 might promote dissociation of cohesin complexes that had somehow evaded cleavage by separase, while hyperphosphorylation of Scc1 (which could also be triggered by Sgo1's departure) might actually facilitate Scc1's cleavage as occurs in both budding yeast [36] and human cells.

The identity of the mitotic kinases responsible for phosphorylation of Scc3-SA2 remains unclear. Because Plk1 is required for dissociation of cohesin from chromosome arms in HeLa cells containing Sgo1 [20], it was surprising that Plk1's depletion did not suppress the precocious loss of cohesion caused by Sgo1 depletion. This finding indicates that protein kinases other than Plk1 can phosphorylate the C terminus of Scc3-SA2. Because Plk1 is necessary for dissociation of cohesin from chromosome arms in the presence of Sgo1 [20] but not in its absence (Figure 7A), Sgo1 would appear to have a role in inhibiting dissociation of cohesin from chromosome arms as well as from centromeres. Though we could not detect Sgo1 associated with chromosome arms, modest amounts residing at this location might (at least partly) inhibit the prophase pathway. We suggest that Sgo1 antagonizes phosphorylation of Scc3-SA2 by several mitotic protein kinases, including Plk1 (Figure 9).

Our study of human Sgo1 has hitherto been confined to HeLa cells. It will be important in the future to analyse its function in other cell types, especially in untransformed cells, and ultimately in real tissues from mice whose Sgo1 gene could be deleted by homologous recombination. It is curious, for example, that Sgo1's likely orthologue in *D. melanogaster,* MEI-S332, is not apparently essential for somatic cell divisions, which contrasts with Sgo1's essential function in HeLa cells. It is conceivable that small changes in the kinetics with which cohesin is dissociated from centromeres in Sgo1/MEI-S332-depleted cells is key to whether a cell is able to undergo anaphase before precocious loss of sister chromatid cohesion triggers the mitotic spindle checkpoint.

One of the most surprising implications of our work is that centromeric sister chromatid cohesion, which has long appeared to be a very stable state and to be the cornerstone (not to mention the most recognisable hallmark) of mitotic chromosomes, only exists because shugoshins protect centromeric cohesin from mitotic protein kinases that maraud mitotic chromosomes and threaten to destroy their integrity. This may have important implications for meiotic cells where protection of cohesin by Sgo1-like proteins (in this case, presumably from attack by separase) is essential for the second meiotic division. Chromosome mis-segregation during meiosis is responsible for trisomies such as Down's syndrome and possibly for age-related infertility and a large fraction of spontaneous abortions [37]. Our work raises the possibility that this could be caused by modest misregulation of protein kinases or phosphatases that affect the phosphorylation of centromeric cohesin. Whether Sgo1 protects centromeric cohesin from separase during meiosis I also by preventing cohesin's phosphorylation is an important question for the future.

## Materials and Methods

### 

#### Antibodies

To generate rabbit anti-Sgo1 antibodies, we synthesized two peptides: one (for which the antibody was termed antibody 94) with the sequence YKEPTLASKLRRGDPFTDL (aa 474–492) from the C terminus of a mouse cDNA (identical to corresponding peptide sequence in human protein) and a second (antibody 95) with the sequence QKRSFQDTLEDOKNRMKEKRNKN (aa 7–29) from the N terminus of a mouse cDNA (the corresponding sequence in human protein contains just four substitutions). Each peptide was conjugated with KLH and injected into rabbits. Sera were taken by a standard scheme, and specific antibodies were obtained by affinity purification using antigenic peptide-conjugated columns. For Western blotting, blots were incubated with the primary antibodies overnight at 4 °C at a 1:500 dilution. For immunofluorescence, cells were incubated with the primary antibody overnight at 4 °C at a dilution of 1:100. Other antibodies used in this study were: mouse anti-Aurora B antibody (anti-AIM-1; BD Transduction Laboratories, San Diego, California, United States); CREST serum (gift from A. Kromminga, Hamburg, Germany); mouse anti-cyclin B1 antibody (Santa Cruz Biotechnology, Santa Cruz, California, United States; #GNS1); rabbit anti-Mad2 serum [38]; mouse anti-myc antibody (clone 4A6, Upstate Cell Signaling Solutions, Charlottesville, Virginia, United States); antibody to phosphohistone H3 (Ser^10^) [P-H3, a mouse monoclonal antibody that detects histone H3 when phosphorylated at serine 10 (Cell Signaling Technology, Beverly, Massachusetts, United States; #6G3)]; rat anti-HP-1β (Serotec Laboratories, Oxford, United Kingdom; #MCA1946); and rabbit anti-SA2 (antibody 466; see [39]).

#### Cell culture, synchronisation, and transfection

HeLa cells were cultured in DMEM supplemented with 10% FCS, 0.2 mM L-glutamine, 100 units/ml penicillin, and 100 μg/ml streptomycin. For synchronisation, HeLa cells were grown in the presence of 1 mM thymidine (Sigma-Aldrich, St. Louis, Missouri, United States) for 12 h, washed with PBS, and grown in fresh medium for 8 h. Cells were then transfected (see below) in serum-free medium (Opti-MEM, Gibco, San Diego, California, United States) supplemented with 0.3% FCS for 4 h, followed by addition of 1 mM thymidine and FCS to a final concentration of 20%. After a further 12 h, cells were again washed in PBS and transferred to fresh medium. Samples were harvested at various time points up to 15 h after the second release, as described below and in the figure legends. Nocodazole and Hesperadin were each used, as indicated, at a final concentration of 100 ng/ml and 100 nM, respectively. Doxycycline-inducible nonphosphorylatable SA2 HeLa cells were established as described in the accompanying paper [17]. For generation of EGFP-H2B stably expressing line, cells were transfected with plasmid encoding EGFP-H2B [40] using FuGene6 reagent (Roche, Basel, Switzerland), and stable expressants were selected in a complete medium containing 2.0 μg/ml blasticidin-S, and were screened by fluorescence microscopy for expression of EGFP-H2B. HeLa cells stably expressing EGFP-CENP-A were established and characterized as described [41]. For flow cytometric analysis, cells fixed in 70% ethanol were washed with PBS and subsequently stained in PI buffer [10 μg/ml propidium iodide, 10 mM Tris-HCl (pH 7.5), 5 mM MgCl_2_, and 200 μg/ml RNase A] for 20 min at 37 °C.

#### RNAi

The oligonucleotide sequence used to target human *Sgo1* (oligo Sgo1.1A) was 5′-CAGU AGAACCUGCU CAGAA-3′. The oligonucleotide sequence used to target human Plk1 cDNA was 5′- CGAGCUGCUUAAU GACGAG-3′ [20]. Synthetic sense and antisense oligonucleotides were purchased from VBC Genomics (Vienna, Austria). For mock transfections, dH_2_O was used instead of siRNA. Transfection of siRNA duplexes was performed at a final concentration of 150 nM. Experiments performed with a second duplex targeted to a different region of human *Sgo1* cDNA (oligo Sgo1.2B) yielded similar results. The oligonucleotide sequence of Sgo1.2B is 5′-GGAUAU CACCAAUGUCUCC-3′.

#### Preparation of HeLa cell extracts

To prepare fractionated HeLa cell extract, cells were harvested by washing in ice-cold PBS and scraping from the plate. Cells were pelleted at 1,200 *g* for 3 min at 4 °C, and washed three times with ice-cold PBS. The cell pellet was resuspended in 200 μl of extraction buffer, which contained 150 mM NaCl, 10 mM NaF, 40 mM β-glycerophosphate, 20 mM HEPES (pH 7.5), 2 mM MgCl_2_, 10 mM EDTA, 0.5% Triton X-100; 10 ml of this buffer was supplemented with one tablet of Complete Protease Inhibitor (Roche) and 1 mM PMSF. Cells were homogenized with ten strokes in a Dounce homogenizer and incubated on ice for 10 min, followed by another ten strokes. Nuclei and insoluble material were pelleted by centrifugation at 13,000 *g* for 10 min at 4 °C. The supernatant was completely removed and denatured by addition of an appropriate volume of 4× Laemmli buffer followed by boiling for 10 min at 95 °C. The pellet was resuspended in 4 volumes of 1× Laemmli buffer, boiled at 95 °C for 10 min, and passed two times through a 27-gauge needle to shear DNA.

#### Immunofluorescence microscopy

For immunostaining, cells were either cultured (and transfected where applicable) directly on glass coverslips, spun onto glass slides using a Cytospin centrifuge (Shandon brand, available from Thermo Electric, Waltham, Massachusetts, United States) for 5 min at 1,500 rpm, or spread on glass slides as described below. Cells were washed with PBS, (in the case of Scc1-myc and SA2-myc staining only, cells were preextracted with 0.1% Triton X-100 in PBS for 2 min followed by PBS wash for 2 min), and fixed in 4% paraformaldehyde in PBS for 15 min. Subsequently, cells were permeabilized with 0.2% Triton X-100 in PBS for 5 min. Cells were blocked in 3% BSA in PBS-T (0.01% Triton X-100) for 1 h prior to incubation with primary antibodies in the same blocking solution. Secondary antibodies were incubated for 1 h at room temperature. The following secondary antibodies conjugated to Alexa Fluor 488 and 568 (Molecular Probes, Eugene, Oregon, United States) were used: goat anti-rabbit, goat anti-mouse, and goat anti-human. DNA was stained by incubating cells in DAPI (1 μg/ml in PBS) for 10 min. Cells were mounted in Vectashield Mounting Medium (Vector Laboratories, Burlingame, California, United States). Images were captured using MetaMorph software (Universal Imaging, Downingtown, Pennsylvania, United States).

#### Chromosome spreads

To prepare chromosome spreads for Giemsa staining, cells were harvested by trypsinization and pretreated with hypotonic buffer containing 40% medium and 60% tap water for 5 min at room temperature. Cells were fixed with freshly made Carnoy's solution (75% methanol and 25% acetic acid), the fixative was changed three times, and the cells were then stored overnight at −20 °C. For spreading, cells in Carnoy's solution were dropped onto glass slides and dried at room temperature. Slides were stained with 5% Giemsa (Merck, Darmstadt, Germany) at pH 6.8 for 10 min, washed briefly in tap water, air-dried, and mounted with Entellan (Merck).

Chromosome spreads for immunostaining were prepared using the method previously described for spermatocyte spreads [42], as adapted for HeLa cells. Briefly, cells were treated with 330 nM nocodazole for 1 h, and mitotic cells were collected by shaking-off. Cells were incubated in a hypotonic buffer [30 mM Tris (pH 8.2), 50 mM sucrose, and 17 mM sodium citrate] for 7 min and then suspended in 100 mM sucrose (pH 8.2). A small volume of the cell suspension was put on a slide glass that had been dipped in fixative [1% paraformaldehyde, 5 mM sodium borate (pH 9.2), and 0.15% Triton X-100] and dispersed on the slide by continuous tilting. After extensive washing with PBS, lysed nuclei were dried and processed for immunofluorescence microscopy.

#### Live cell imaging

Cells were grown and synchronised in Lab-Tek chambered cover glasses (Nunc, Roskilde, Denmark) and transfected with siRNA as described in Figure 1. At 6 h after the release from the second thymidine block, medium was changed to CO_2_-independent medium without phenol red, and the chambers were sealed with silicone grease. Three-dimensional image stacks of live cells were captured over time using either Zeiss 510 confocal microscope (Figure 4A and 4C; Zeiss, Oberkochen, Germany) or Olympus BX51 fluorescence microscope (Figures 4B and 8D; Olympus, Tokyo, Japan).

## Supporting Information

Figure S1 Cloning and Expression Analysis of hsSgo1 and mmSgo1 (A) The expression pattern and primary structures of human and mouse Sgo1 (hsSgo1 and mmSgo1, respectively) transcription products were investigated. Total RNA from HeLa cells, mouse testis and mouse thymus were prepared using TRIzol reagent (Invitrogen, Carlsbad, California, United States) according to the manufacturer's instructions. First-strand cDNA was synthesized by SuperScript II reverse transcriptase (Life Technologies, Carlsbad, California, United States) primed by an oligo dT primer, and PCR was performed using the Expand High Fidelity PCR system (Roche). For *mmSgo1* cDNA amplification, we designed primers 5′- CTGAGTGGCCGAGATGAATTTCAC-3′ and 5′- TGTCCAAGAGACCCTCCTGATCAG-3′ according to GenBank cDNA NM_028232.
We obtained two major bands at 0.9 and 1.8 kb [lane 1 (testis) and lane 2 (thymus)] and cloned them into the pGEM-T vector (Promega, Madison, Wisconsin, United States). Multiple clones were sequenced and PCR error-free sequences *mmSgo1A* and *mmSgo1B* were obtained. Note that when the secondary PCRs were done using the PCR reaction above as templates and primers 5′- AATCTATGACCCACCTGCCTTAGC-3′ and 5′- GGTTCTGCCCATTGTGCACTGTCT-3′, we saw three minor bands between 0.9 and 1.5 kb (unpublished data), suggesting multiple species of splicing variant.
For *hsSgo1* cDNA cloning, we had found two potential splicing variants in the ENSEMBL database (BC001339 and BC017867) that have different 3′ sequences. When primers 5′- CTGGAGAGCTTCGAAGAGCCTTGA-3′ and 5′- CCTCTCCTGAAGCAACAGAAAGAG-3′ (designed to amplify products from BC001339) were used, 0.9- and 1.0-kb bands were detected (lane 3), and *hsSgo1A–hsSgo1D* cDNAs were obtained. When primers 5′- CTGGAGAGCTTCGAAGAGCCTTGA-3′ and 5′- CTGAGTGAAACAGACTGTCAACAC-3′ (designed to amplify products from BC017867) were used, six bands between 0.9 and 2.0 kb were detected (lane 4), and cDNA clones obtained from those bands were named *hsSgo1E–hsSgo1H* and *hsSgo1J–hsSgo1L*. We could not obtain a clone that encodes the identical open reading frame encoded by BC001339.
(B) To examine the expression of *Sgo1* in HeLa cells and various other human and mouse tissues, we performed Northern analysis. Total HeLa RNA was prepared as described in (A), and 20 μg was run on a formaldehyde agarose gel and RNA blots were prepared. A blot was probed with a [^32^P]-labelled probe (probe H1) generated against the common N terminus region of *hsSgo1* [nucleotides (nt) 1–647 of hsSgo1E). Many bands, although faint and ambiguous, were seen (lane 1). We could not assign each signal to a specific cloned cDNA variant; however, these data support the notion that many species of splicing variant are expressed in HeLa cells. When the blot was probed by another probe (probe H2) that is designed to hybridize to the central region specific for *hsSgo1E* and *hsSgo1F* cDNAs (nt 665–1454 of hsSgo1E), a signal at ∼2.2 kb was seen (lane 2). Using two probes of *hsSgo1,* we could confirm that the two relatively long transcripts, *hsSgo1E* and *hsSgo1F,* that possess the central region, and the other shorter transcripts that lack the central region, are expressed in HeLa cells.
(C) The tissue distribution of *hsSgo1* mRNA was studied using a commercially available Multi Tissue Northern blot (BD Clontech, Palo Alto, California, United States). Multiple, relatively strong, signals were detected in testis, among other organs, on both blots probed by H1 and H2 (upper left blot and lower left blot, respectively). This higher expression of *Sgo1* in testis might reflect the fact that testis contains a higher fraction of proliferating cells. Expression analysis of mouse Sgo1 was also performed in parallel. Both a probe against the common N-terminal region of mmSgo1 (probe M1, corresponding to nt 1–610 of *mmSgo1A*) and an mmSgo1A-specific probe (probe M2, corresponding to nt 704–1339 of *mmSgo1A*) detected potential mmSgo1 messages in spleen and testis (upper right blot by probe M1, and lower right blot by probe M2, respectively). Mouse Sgo1 messages in spleen were more abundant than in testis, but human Sgo1 message was not detected in human spleen. The biological significance of the different levels of Sgo1 expression observed between human and mouse spleen is unclear. The tested polyA^+^ RNAs are from spleen (SP), thymus (TH), prostate (PR), testis (TE), ovary (OV), small intestine (SI), colon (CO), peripheral blood leukocyte (PL), heart (HE), brain (BR), lung (LU), liver (LI), skeletal muscle (SM) and kidney (KI). In conclusion, we cloned multiple splicing variants of hsSgo1s and mmSgo1s from HeLa cells and mouse organs. The presence of a variety of messages was also confirmed. However, the functional difference between and biological meaning of such variation remains unknown and requires further study.
(D) Structures of cDNAs for *hsSgo1A–hsSgo1H* and *hsSgo1J–hsSgo1L*.
(E) Predicted structures of proteins depicted as cDNAs in (D).
(F) Structures of cDNAs for *mmSgo1A* and *mmSgo1B*.
(G) Predicted structures of proteins depicted as cDNAs in (F).
(2.7 MB EPS).

Figure S2 Sgo1 Antibody Characterisation Identical Sgo1 localisation patterns were obtained by staining cells with two peptide antibodies raised against different regions of the protein (see Materials and Methods for details).
(A) HeLa cells grown on glass coverslips were paraformaldehyde-fixed and costained with Sgo1 antibody 94 (directed against C-terminal peptide, shown in green) and CREST antiserum (shown in red) to label kinetochores. Five different stages of mitosis are illustrated as indicated.
(B) As (A), except cells were costained with Aurora B antibody (shown in red) to label inner centromere.
(C) As (B), except Sgo1 was stained with antibody 95 (directed against N-terminal peptide, shown in green).
(10 MB EPS).

Figure S3 Precocious Sister Separation Is Specific to Sgo1 siRNA Treatment Synchronised HeLa cells were transfected as described in Figures 1 and 3 with siRNA oligonucleotides directed against the following proteins: Aurora B, condensin subunits Cap-D2, Cap-D3, Cap-G, Cap-G2 and Smc2, Cyk4, Ect2, Lamin A, myosin heavy chain Type II (MHC), MKLP1, RhoA, and Sgo1 (two oligonucleotide targets; see Materials and Methods). Cells were harvested 11 h after the second thymidine release and analysed as described for Figure 3A and 3B. The histogram indicates the frequency of each observed category as a percentage of total cells, such that the sum of each column represents the mitotic index. The separated sisters category (represented by the red bars) combines the three categories illustrated in Figure 3B, part e–g.
(1.3 MB EPS).

Figure S4 No Reduction of Cohesin on Prophase Chromosomes in Sgo1-Depleted Cells HeLa cells expressing Scc1-myc were mock-treated or Sgo1 siRNA-transfected and harvested as described for Figure 5, cells spun down on glass slides, preextracted prior to fixation, and analysed by immunofluorescence microscopy using myc antibody. Cells were costained with P-H3. Prophase cells, identified by positive P-H3 staining with intact nuclear envelope, were measured for their fluorescence intensities of Scc1-myc and P-H3 using Image-J software. The ratio of fluorescence intensities of myc signals to P-H3 signals was calculated.
(583 KB EPS).

Figure S5 Induction of SA2-myc Expression upon Doxycycline Treatment A sample of the cells from the experiment described in Figure 8B were spun onto glass slides and processed for immunofluorescence, staining with myc antibody as described (but cells were not preextracted with 0.1% Triton X-100 prior to fixation). In uninduced cells, a moderate level of leaky expression of the tagged protein is observed in a small number of cells (photomicrographs a, b, e, and f). In induced cells, a high level of the tagged protein is observed in the vast majority of cells (photomicrographs c, d, g, and h). Anti-myc staining of HeLa cells which do not contain any myc-tagged gene expression cassette (photomicrograph i).
(4.7 MB TIF).

### Accession Numbers

The NCBI GeneID (http://www.ncbi.nlm.nih.gov/) and ENSEMBL (http://www.ensembl.org/) accession numbers of the genes discussed in this paper are *hsSgo1* (GeneID 151648; ENSEMBL gene ENSG00000129810) and *mmSgo1* (Gene ID 72415; ENSEMBL gene ENSMUSG00000023940). The pending GenBank/EMBL/DDBJ accession numbers of the cDNA sequences discussed in Figure S1 are *hsSgo1A* (AB193056), *hsSgo1B* (AB193057), *hsSgo1C* (AB193058), *hsSgo1D* (AB193059), *hsSgo1E* (AB193060), *hsSgo1F* (AB193061), *hsSgo1G* (AB193062), *hsSgo1H* (AB193063), *hsSgo1J* (AB193064), *hsSgo1K* (AB193065), *hsSgo1L* (AB193066), *mmSgo1A* (AB193067), and *mmSgo1B* (AB193068).

## Abbreviations

APC/C: anaphase-promoting complex or cyclosome
CREST: calcinosis
DAPI: 4′,6′-diamidino-2-phenylindole
EGFP: enhanced green fluorescent protein
NEBD: nuclear envelope breakdown
nt: nucleotide(s)
P-H3: phosphohistone H3 (Ser^10^)
RNAi: RNA interference
siRNA: small interfering RNA
